# Renal Cancer Detection: Fusing Deep and Texture Features from Histopathology Images

**DOI:** 10.1155/2022/9821773

**Published:** 2022-03-28

**Authors:** Jianxiu Cai, Manting Liu, Qi Zhang, Ziqi Shao, Jingwen Zhou, Yongjian Guo, Juan Liu, Xiaobin Wang, Bob Zhang, Xi Li

**Affiliations:** ^1^PAMI Research Group, Department of Computer and Information Science, Avenida da Universidade, University of Macau, Taipa, Macau, China; ^2^Department of Radiology, The Second Affiliated Hospital of Guangzhou Medical University, Guangzhou, China

## Abstract

Histopathological images contain morphological markers of disease progression that have diagnostic and predictive values, with many computer-aided diagnosis systems using common deep learning methods that have been proposed to save time and labour. Even though deep learning methods are an end-to-end method, they perform exceptionally well given a large dataset and often show relatively inferior results for a small dataset. In contrast, traditional feature extraction methods have greater robustness and perform well with a small/medium dataset. Moreover, a texture representation-based global approach is commonly used to classify histological tissue images expect in explicit segmentation to extract the structure properties. Considering the scarcity of medical datasets and the usefulness of texture representation, we would like to integrate both the advantages of deep learning and traditional machine learning, i.e., texture representation. To accomplish this task, we proposed a classification model to detect renal cancer using a histopathology dataset by fusing the features from a deep learning model with the extracted texture feature descriptors. Here, five texture feature descriptors from three texture feature families were applied to complement Alex-Net for the extensive validation of the fusion between the deep features and texture features. The texture features are from (1) statistic feature family: histogram of gradient, gray-level cooccurrence matrix, and local binary pattern; (2) transform-based texture feature family: Gabor filters; and (3) model-based texture feature family: Markov random field. The final experimental results for classification outperformed both Alex-Net and a singular texture descriptor, showing the effectiveness of combining the deep features and texture features in renal cancer detection.

## 1. Introduction

Histopathology images contain markers of disease progression and morphological information, supplying a clear view of the tiny structures in tissue such that it is considered as the final diagnosis for cancer subtype [1, 2]. That being said, most hospitals lack pathologists; take Germany as an example, where a large shortage of pathologists in Germany could lead to a bottleneck in the health system [3]. The classification of histopathology images by pathologists is a very challenging task. First, there is interobserver discordance between pathologists due to their different capabilities and experiences. Second, the complicity of histopathology images makes diagnosis time-consuming. Many computer-aided diagnosis (CAD) systems have been proposed to overcome these difficulties by extracting the features from histopathology images to identify subtle differences between clinical categories [4, 5], such as breast histopathology images [2], lung histopathology images [6], and kidney histopathology images [7].

Renal cancer (RC) is one of the worst cancers in the world. The American Cancer Society indicated that 76,080 new cases and 13,780 deaths will occur in 2021 [8]. Though renal cancer develops slowly, early treatment can improve the cure rate and survival time. There has been a substantial amount of research applying deep learning methods to classify renal cancer from histopathology images, of which deep learning methods have worked well given a large dataset. Tabibu et al. [7] used convolutional neural networks (CNNs) with a whole-slide image dataset with over 1500 samples to classify Renal Cell Carcinoma (RCC) subtypes and predicted the survival outcome from digital histopathological images, achieving an accuracy of 94.07%. Fenstermaker et al. [9] randomly selected over 15000 patches with a size of 1024 × 1024 pixels and achieved an accuracy of 99.1% for the classification of normal parenchyma and RCC using a CNN model. Fenstermaker et al. [9] developed deep convolutional neural networks (DCNNs) to diagnose renal cancers using a dataset of about 30000 whole slide histopathology images from The Cancer Genome Atlas (TCGA) and successfully detected malignancy with an AUC of 0.964-0.985.

However, from those literatures mentioned above, we know that deep learning methods require a large dataset to reach a relatively high accuracy, which can be difficult to obtain due to the scarcity of public medical datasets. Besides this, due to the intrinsic complexity of histopathology images, there are very subtle differences between images in different categories. If relying on deep learning only, misjudgements are unavoidable. At the same time, in histopathology images, there are repetitive patterns which can be particularly suited for texture analysis. Texture is generally characterized by homogenous areas with properties related to scale and regular patterns, texture analysis plays an importance role in many medical image analysis [10], such as medical image classification [11], medical image segmentation [12], and medical images retrieval [13]. Given a smaller histopathology image dataset, traditional texture feature extractors can reach a reasonable result. Alhindi et al. [14] compared local binary pattern (LBP), histogram of gradients (HOG), and deep features (VGG16) for classification of a smaller histopathology images dataset containing less than 1000 samples. The result showed that LBP achieved the highest accuracy of 90.52% with the support vector machine (SVM), which is lower than the accuracies that used a large dataset as we mentioned above, but better than the deep learning method. In [15], the HOG feature from gastric cancer histopathology images was extracted from normal, benign, and malignant gastric images. The accuracy rate of this work was 100%, which is quite impressive.

**Figure 1:**
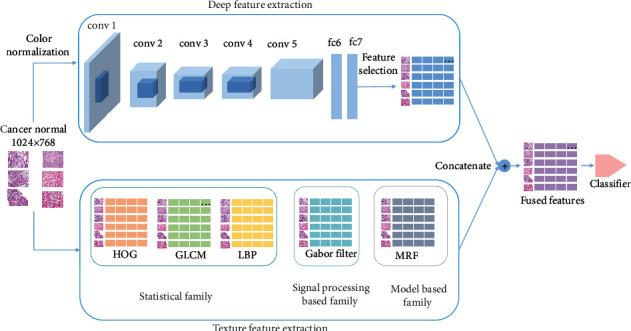
The procedure of the proposed model. Images of 1024 × 768 pixels in size are color normalized, where a 4096-dimensional feature vector is extracted from the deep model for each image. Next, we select a 100-dimensional vector by feature selection for each image. Finally, the deep features with texture features are combined for classification.

Currently, deep learning methods are the most frequently studied and successful type of machine learning methods, and the adoption of deep learning in histopathology images [2, 6, 7, 16] has demonstrated its usefulness. While deep learning methods generate an abstract representation that is learned in the hidden layers of the neural network, traditional texture feature extractors generate more mathematically solid features that are particularly suitable for histopathology images and can reach a reasonable result without a large dataset. However, there exist few literatures on renal cancer detection using texture features. To combine the advantages of both the deep learning method and traditional methods, we proposed a classification model shown in Figure 1, which can be used to improve the classification accuracy for renal cancer detection. For the deep learning method, we utilized Alex-Net to extract robust deep features without experiencing overfitting. For traditional methods, we employed five texture descriptors from three families as shown in Table 1 to complement Alex-Net. The contributions of this work are as follows: (1) we proposed a model consisting of color normalization, deep features, texture features, and feature selection to do renal cancer detection; (2) we applied our model on a small histopathology image dataset collected by a hospital, where the results of the proposed model outperformed either the deep learning method or a single traditional texture feature method.

The rest of this paper is organized as follows.  Section 2 presents Alex-Net along with five texture feature extraction methods with our applied fusion method.  Section 3 explicitly shows the experiment results of our model for renal cancer detection and discusses the results and outlines our findings.  Section 4 summarizes the research.

## 2. Materials and Methods

### 2.1. Dataset

In this paper, the dataset we used was provided by The Second Affiliated Hospital of the Guangzhou Medical University. It contains 93 RC and 150 patients with healthy kidneys who were enrolled and treated between the year 2010 and the year 2019. For each patient, there are an average of two histopathology images with a size of 1024 × 768 and some images are not in good quality to be included. These histopathology images have been manually diagnosed by multiple doctors. For the purpose of generalizability, we performed rotation and flipping on each image. After preprocessing, we set the proportions of training and testing as 7 : 3; the statistics are as Table 2 shows.

### 2.2. Preprocessing

**Figure 2:**
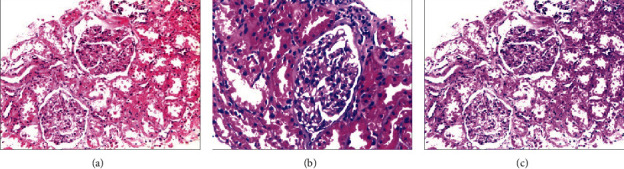
An example of color normalization: (a) is the source image; (b) is the target image; there is color variation between (a) and (b); (c) is the resultant image after color normalization.

In a histopathology image, nuclei are dyed purple, while the other structures are pink. Different structures are distinguishable for the use of manual or automated analysis. However, the color variants due to the preparation of tissue sections like difference of the staining procedure make those analyses difficult. To improve the generalizability of the model confronting data with difference in color styles, we used Structure-preserving Color Normalization (SPCN) [17], which was proposed by Vahadane et al. to control the color variation and contrast enhancement by preserving the structure of the histopathology images. Stain separation is the key step of color normalization, where it first casts the stain separation problem as a nonnegative matrix factorization (NMF) to which they add a sparseness constrain and refer to it as sparse nonnegative matrix factorization (SNMF) with a cost function shown in Equation (1). With the SNMF, for a given source image *s* and a target image *t*, their color appearances and stain density maps can be estimate by factorizing *V*_*s*_ into *W*_*s*_*H*_*s*_ and *V*_*t*_ into *W*_*t*_*H*_*t*_. Then, a scaled version of the density map of source *H*_*s*_ is combined with the color appearance of the target *W*_*t*_ instead of the source *W*_*t*_ to generate the normalized source image, which can be described as Equations (2)–(4).

(1) $$\begin{array}{c} \min_{W,,H}\frac{1}{2}{\left\|V-WH\right\|}_{F}^{2}+\lambda \overset{r}{\underset{j=1}{\sum }}{\left\|H\left(j,:\right)\right\|}_{1}\;\text{ such that }W,H\geq 0,{\left\|W\left(,j\right)\right\|}_{2}^{2}=1, \end{array}$$

(2) $$\begin{array}{c} H_{s}^{\mathrm{norm}}\left(j,:\right)=\frac{H_{s}\left(j,:\right)}{H_{s}^{\text{RM}}\left(j,:\right)}H_{t}^{\text{RM}}\left(j,:\right),j=1,\cdots ,r, \end{array}$$

(3) $$\begin{array}{c} V_{s}^{\mathrm{norm}}=W_{t}H_{s}^{\mathrm{norm}}, \end{array}$$

(4) $$\begin{array}{c} I_{s}^{\mathrm{norm}}=I_{0}\exp\left(-V_{s}^{\mathrm{norm}}\right), \end{array}$$

where *H*_*i*_^RM^ = RM(*H*_*i*_) ∈ *R*^*r* *x* 1^, *i* = (*s*, *t*) and RM(⊙) compute robust pseudomaximum of each row vector at 99%.  Figure 2 shows an example of color variation and color normalization.

### 2.3. Alex-Net

The deep learning features were extracted by Alex-Net [18], a classical convolution neural network, and have been widely applied in various medical image analysis tasks such as cancer detection [19] and lesion segmentation [20]. Nawaz et al. [19] fine-tuned Alex-Net by changing and inserting the input layer convolutional layers and fully connected layer, achieving a patch and image-wise accuracy of 75.73% and 81.25%, respectively, given a dataset consisting of 400 images (which is not high). Titoriya and Sachdeva [21] used the AlexNet model with the BreakHis dataset [22], and the training model achieved spectacular classification accuracy ranging between 93.8% and 95.7% with a dataset of about 8000 images.

**Figure 3:**
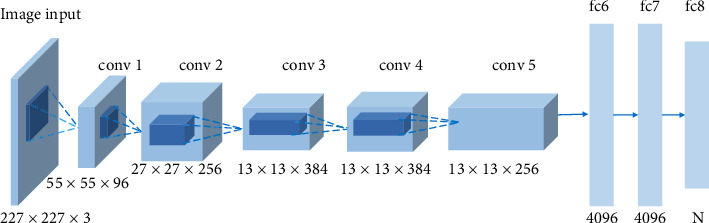
The simplified architecture of Alex-Net.

The network consists of eight layers. The first five layers are convolutional layers, the last layers are fully connected layers, and the output of the last fully connected layer is passed to a softmax classifier; the simplified architecture is shown in Figure 3. There are several main characteristics of the network. First, it successfully used rectified linear units (ReLU) shown in Equation (5) as the activation function and verified that its effectiveness surpassed sigmoid in a deep network. Second, it used dropout to randomly ignore some neurons during training to avoid overfitting of the model. Moreover, it also used data augmentation consisting of horizontal reflection to overcome the problem of overfitting. Third, it used overlapped max pooling to avoid the blurring effect of average pooling. Besides this, it proposed local response normalization (LRN), which creates a competition mechanism for the activity of the local neurons so that the value with a larger response becomes relatively large, and other neurons with smaller feedback are inhibited, enhancing the generalization ability of the model. The response-normalized activity *b*_*x*,*y*_^*i*^ is given by Equation (6).

(5) $$\begin{array}{c} R\left(z\right)=\max\left(0,z\right), \end{array}$$

(6) $$\begin{array}{c} b_{x,y}^{i}=\frac{a_{x,y}^{i}}{{\left(k+\alpha \sum_{j=\max\left(0,i-\left(n/2\right)\right)}^{\min\left(N-1,i+\left(n/2\right)\right)}{\left(a_{x,y}^{j}\right)}^{2}\right)}^{\beta }}. \end{array}$$

### 2.4. Texture Feature Extraction

The eight texture extractors are described in this subsection. First, the three methods (IGH, GLCM, and LBP) from the statistical texture feature family are given. Afterwards, Gaussian filter from the transform-based family is described. Finally, MRF coming from the model-based family is introduced.  Table 1 lists the five methods from the three families.

### 2.5. Statistical Texture Feature Family

The statistical texture feature descriptors are based on the statistical properties of the spatial distribution of the grey levels [23–25]. The statistical characteristics include the first-order (one pixel), second-order (two pixels), and higher-order (three or more pixels) statistics. The first-order statistics estimate properties of one pixel value, whereas second- and higher-order statistics evaluate properties of the spatial interaction between two and more image pixels [24]. To explore the various order statistics of kidney histopathology images, HOG (first order), GCLM (second order), and LBP (higher order) are used.

#### 2.5.1. Histogram of Oriented Gradients (HOG)

**Figure 4:**
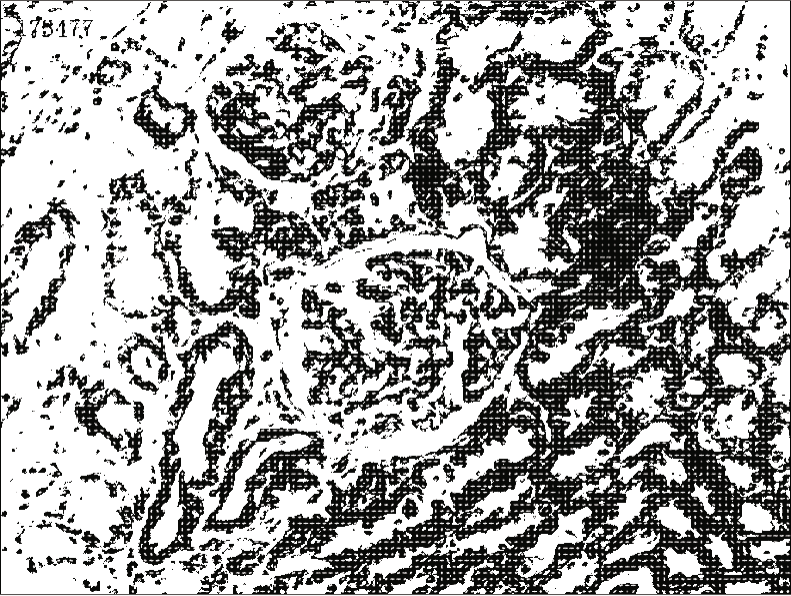
An example of plotting HOG features over the original image.

This feature is a feature descriptor used for object detection in computer vision and image processing. It composes the features by calculating and counting the histogram of the gradient direction of the local area of the image. HOG feature combined with a SVM classifier has been widely used in image recognition, especially in pedestrian detection [26]. It operates on the local grid cell of the image, which enables it to maintain a good invariance to the geometric and optical deformation of the image [27]. Since there is large randomness of viewing angles from the process of creating histopathology images, the HOG feature is particularly suitable for the feature extraction of histopathology images.  Figure 4 is an example of plotting the HOG features over the original image.

HOG feature extraction steps are as shown [27].

1. Normalize the image: convert the input image to a grayscale image and use the Gamma filter method to perform global normalization on the grayscale image. The purpose is to avoid the influence of noise in the image
2. Calculate the gradient value and direction of the image to describe the structure and shape of the image and eliminate the interference of noise. The formulas are as follows:

(7) $$\begin{array}{c} G_{x}\left(x,y\right)=H\left(x+1,y\right)-H\left(x-1,y\right), \end{array}$$

(8) $$\begin{array}{c} G_{y}\left(x,y\right)=H\left(x,y+1\right)-H\left(\text{x},y-1\right), \end{array}$$

(9) $$\begin{array}{c} G\left(x,y\right)=\sqrt{G_{x}{\left(x,y\right)}^{2}+G_{x}{\left(x,y\right)}^{2},} \end{array}$$

(10) $$\begin{array}{c} \alpha \left(x,y\right)=\arctan\left[\frac{G_{y}\left(x,y\right)}{G_{x}\left(x,y\right)}\right], \end{array}$$

where *G*(*x*, *y*), *G*_*x*_(*x*, *y*), *G*_*y*_(*x*, *y*), and *H*(*x*, *y*) are the gradient value of the current pixel, horizontal gradient, vertical gradient, and pixel value and *α*(*x*, *y*) is the gradient direction.

1. Divide the image into cell units and construct a gradient histogram. The cell size will affect the encoding of the feature vector. If the cell size is too large, it will lead to incomplete coding of the feature information; if the cell size is too small, it will lead to an increase in the time complexity
2. Combine the preset number of cells into a block; obtain the normalized gradient histogram within the block. For example, for the size of a 16 pixel × 16 pixel image, divided into 16 cells with a size of 4 pixels × 4 pixels, each adjacent 4 cells form a normalized block, each cell has 9 features, and the step size of the sliding window is 4 pixels; that is, each block corresponds to a 36-dimensional feature vector
3. Concatenate the features of all blocks to get the HOG features of the image

#### 2.5.2. Gray-Level Cooccurrence Matrix (GLCM)

**Figure 5:**
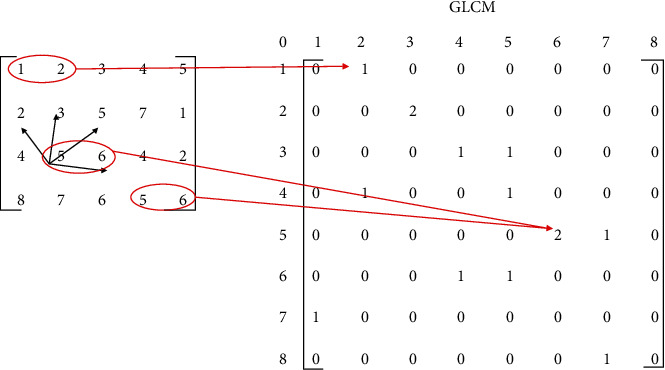
An example of GLCM generation. In the GLCM, element (1, 1) is equal to 1 because there is only one instance in the input image where two horizontally adjacent pixels have the values 1 and 1, the same for the element (5, 6).

GLCM is a well-known texture analysis method by extracting the second-order statistical texture features [28–30]. Each element *P*(*i*, *j* | *d*, *θ*) in GLCM corresponds to the number of occurrences of the pairs of gray levels *i* and *j* which are at a distance *d* apart in the direction of *θ*.  Figure 5 shows an example of the computation for GLCM [31] . Here, there is an image with 8 gray levels, where the size of GLCM is 8 × 8. When *d* = 1 and *θ* = 0, gray level (1, 2) appears once, meaning that the element *P*(1, 2) in GLCM equals 1, while the gray level (5, 6) appears twice and the element *P*(5, 6) in GLCM is set as 2. Once the matrices are computed, various properties can be extracted to represent the texture of the image. In this paper, four properties are extracted (in what follows, the image has *N* discrete intensity levels):

(11) $$\begin{array}{c} \text{Contrast}=\overset{N-1}{\underset{i,j=0}{\sum }}iP_{i,j}{\left(i-j\right)}^{2}, \end{array}$$

(12) $$\begin{array}{c} \text{Correlation}=\overset{N-1}{\underset{i,j=0}{\sum }}ijP_{i,j}\mu_{1}\mu_{2}, \end{array}$$

(13) $$\begin{array}{c} \text{Energy}=\overset{N-1}{\underset{i,j}{\sum }}{P_{i,j}}^{2}, \end{array}$$

(14) $$\begin{array}{c} \text{Homogeneity}=\overset{N-1}{\underset{i,j=0}{\sum }}\frac{P_{i,j}}{1+{\left(i-j\right)}^{2}}, \end{array}$$

where contrast evaluates the local variations in the matrix, correlation measures the joint probability occurrences of the pairs, and energy is the sum of squared elements in the matrix, which provides information on image homogeneity; a low value means the probabilities of the gray-level pairs are rather similar and high values otherwise. Besides that, homogeneity estimates the proximity of the distribution of elements in the matrix.  Table 3 is an example of the four properties of kidney histopathology images from a normal and RC sample.

#### 2.5.3. Local Binary Pattern (LBP)

**Figure 6:**
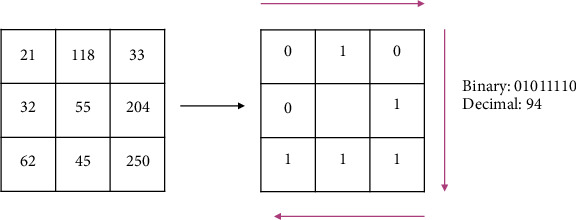
An example of LBP feature value of a pixel.

**Figure 7:**
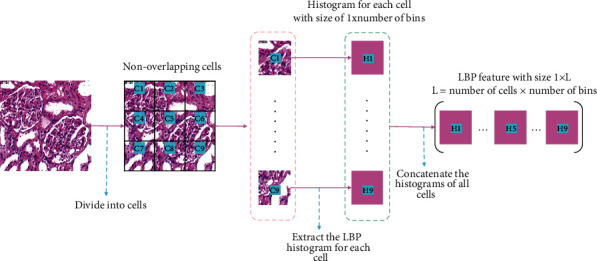
An example of extracting LBP features of an image.

LBP was introduced in [32] to characterize texture features presented in grayscale images, and it has been widely used in many fields of computer vision due to the simple calculation and its good performance, especially in face recognition [33] and object detection [34]. First, the input image is divided into nonoverlapping cells, and histograms are extracted from each of those cells, respectively. Taking a window with size of a 3 × 3 as shown in Figure 6, the threshold is the gray scale of the center pixel; compare its 8 neighbors with the threshold. If the neighbor is large, its value is set as “1,” otherwise it is “0.” From left to right and top to bottom, an 8-bit binary number is generated and converted to decimal as the LBP value of the center pixel. Over the cell, a histogram is computed based on the frequency of each decimal number. Then, the histograms are concatenated into the LBP features of the image to represent the image, where the size of the LBP features depends on the number of cells and the number of bins of the histograms.  Figure 7 is an example of extracting the LBP features from an image.

### 2.6. Transform-Based Texture Feature Family

Transform-based texture descriptors commonly use linear transformers, filters, or filter banks to transform images into another space to distinguish texture more easily in the new space [10]. The Gabor filter is a very useful linear filter used for texture analysis [35].

#### 2.6.1. Gabor Filter

**Figure 8:**
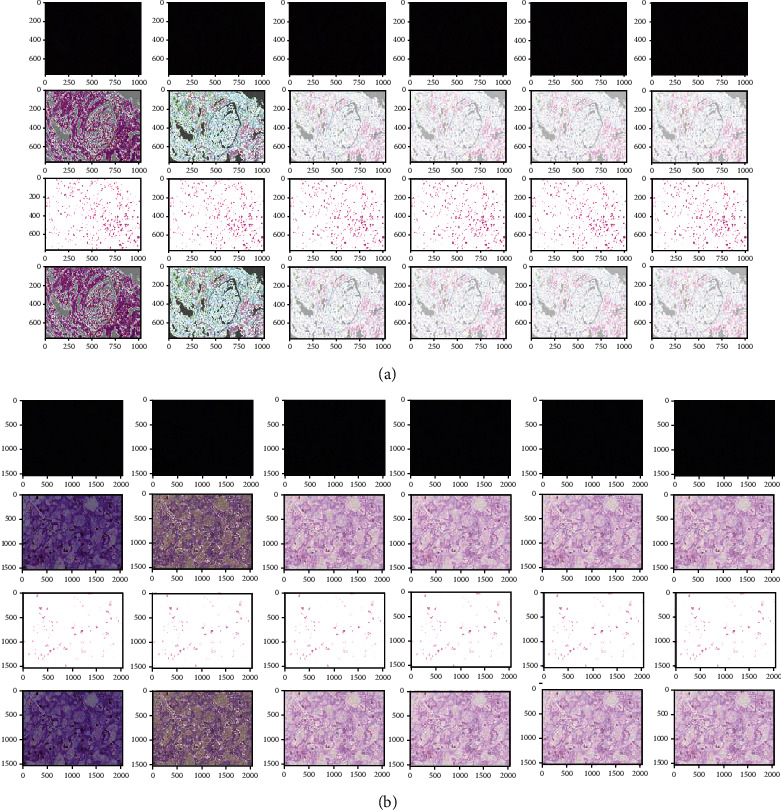
(a) An example of Gabor filter outputs of a healthy kidney histopathology image. (b) An example of Gabor filter outputs of a RC kidney histopathology image.

A Gabor filter has frequency and direction that are similar to the human visual system, which makes it very helpful in image processing, especially in face recognition [36]; a 2-D Gabor filter is defined as Equation (15) [37]. In the original spatial domain, a Gabor kernel is the result of a Gaussian kernel and sine wave modulation, and images are filtered by the real parts of the Gabor filter kernels. Then, the mean and variance of the filtered images are used as texture features for image classification. For this paper, we set various filter sizes to extract the texture feature of the histopathology images.  Figure 8 is an example of Gabor output from a healthy and RC kidney histopathology images with the filter size being 24.

(15) $$\begin{array}{c} \text{G}\left(x,y\right)=e^{\left(x^{2}+{ɣ}^{2}y^{2}\right)/-2\sigma^{2}}\cos\left(2\pi \frac{x^{\prime }}{\lambda }\right), \end{array}$$

where *x*′ = *x* · con*θ* + *y* · sin*θ*, *y*′ = *x* · sin*θ* + *y* · cos*θθ*, *σ* is the variances, *θ* is the wavelength, *ɣ* is the aspect ratio of the sinusoidal function, and *θ* is the orientation.

### 2.7. Model-Based Texture Feature Family

Model based methods construct an image model and use the parameters of the model as its texture features, where its main goal is to optimize the parameters. There are several commonly used methods such as mosaic models and random field models [10]. MRF as a typical method of a random field model is used to extract texture features from kidney histopathology images.

#### 2.7.1. Markov Random Field (MRF)

*X*_*n*_ is a Markov random process if its different conditions confirm Markov chain and satisfy Equation (16), which implies that each element is only related to its neighbors and not influenced by the nonneighboring elements. Markov chains that are extended to multiple dimensions are called MRF [38]. MRF has been applied in many fields of image processing such as segmentation [39] and classification [40], with its main advantage being that it provides the interrelationship of the related random variables in the expression space and makes full use of the statistical dependence of the neighbor pixels.

(16) $$\begin{array}{c} P\left(X_{n}=x_{n}\;|\;X_{nk}=x_{k},k\neq n\right)=P\left(X_{n}=x_{n}\;|\;X_{n-1}=x_{n-1},X_{n+1}=x_{n+1}\right). \end{array}$$

### 2.8. Proposed Method

To exclude any redundant information from the deep learning features, we applied feature selection before classification based on the differences between the positive and negative labels (RC and healthy). The difference of the *k*th feature diff_*k*_ is calculated as

(17) $$\begin{array}{c} {\text{diff}}_{k}=\left|\frac{1}{N_{\text{pos}}}\underset{i\in \text{pos}}{\sum }v_{i,k}-\frac{1}{N_{\text{neg}}}\underset{i\in \text{neg}}{\sum }v_{i,k}\right|, \end{array}$$

where *k* ranges from 1 to 4096, *N*_pos_ and *N*_neg_ are the number of positive and negative images in the training set, and *v*_*i*,*k*_ is the *k*th dimensional feature of the *i*th image. Feature components are then ranked from the largest diff_*k*_ to smallest, and the top 100 feature components are selected [41]. We terminated the training after 5 epochs when the validation accuracy did not improve.

In this subsection, we proposed a model to tackle the issue of RC detection. The detailed steps of our framework are shown in Figure 1. After image preprocessing, feature extraction, feature selection, and feature fusion, we can eventually classify RC from healthy kidneys using histopathology images.

## 3. Results and Discussion

In this section, we validated the proposed model on the dataset mentioned in Section 2.1. The experiments were implemented in MATLAB 2020a with an Intel Core I7 computer processor, 16 GB of RAM, and a Windows 10 system. Three traditional classifiers of LR, SVM, and RF were chosen to detect RC based on the merged features, and we repeated the experiment for ten times and got the average as the final result. For LR, the penalty is set as “l2” and *C* equals to 1.0, while the linear kernel function is used and *C* is equal to 0.025 in SVM. In terms of RF, the criterion is entropy, and the maximum depth of the tree is equal to 3. We adopted accuracy, precision, recall, and F1 score as evaluation metrics for the proposed model, defined as follows:

(18) $$\begin{array}{c} \text{Accuracy}=\frac{\text{True Pos}.+\text{True Neg}.}{\text{All data number}}, \end{array}$$

(19) $$\begin{array}{c} \text{Precision}=\frac{\text{True Pos}.}{\text{True Pos}.+\text{False Neg}.}, \end{array}$$

(20) $$\begin{array}{c} \text{Recall}=\frac{\text{True Neg}.}{\text{True Neg}.+\text{False Pos}.}, \end{array}$$

(21) $$\begin{array}{c} \text{F}1\text{ score}=2*\frac{\text{Recall}*\text{Precision}}{\text{Recall}+\text{Precision}}, \end{array}$$

where True Pos. is the class of correctly classified normal kidney images and True Neg. represents the class of correctly classified RC histopathology images. False Pos. is the incorrectly classified normal kidney images, and False Neg. is the incorrectly classified RC images.

### 3.1. Deep Feature Results

**Figure 9:**
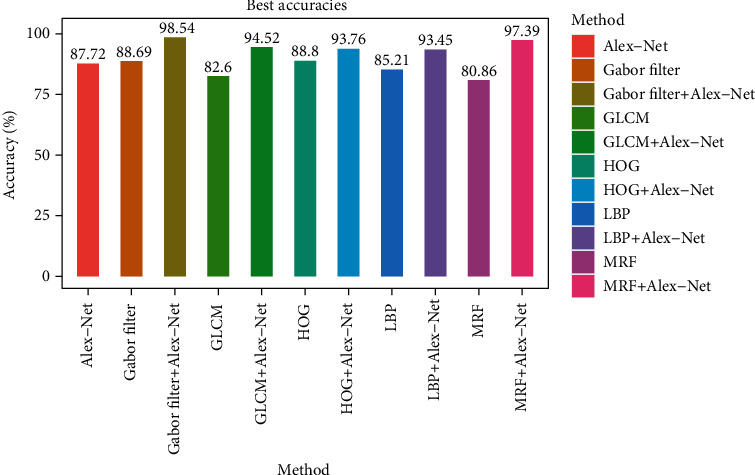
Best accuracies of Alex-Net, different texture feature extractors, and the proposed model.

For Alex-Net, we fine-tuned the training parameters and trained Alex-Net by ImageNet. Then, we extracted the features from the histopathology images via the “fc7” layer and obtained a 4096-dimensional vector for each image [42]. We terminated the training after 20 epochs when the validation accuracy did not improve. An accuracy of 87.72% with a precision of 81.86%, a recall of 98.25%, and a F1 score of 88.89% was obtained as shown in Figure 9.

### 3.2. Statistical Texture Feature Family Results

#### 3.2.1. HOG Results

**Figure 10:**
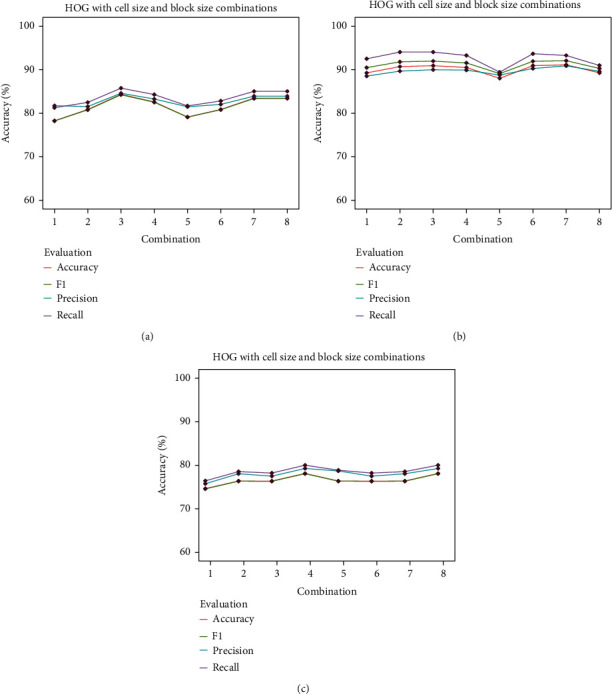
HOG with different cell size and block size combinations.

In HOG, we analysed a range of combinations of cell sizes and block sizes (refer to Table 4) for renal cancer detection [43]. As Figure 10(a) shows the results of HOG using LR, the best accuracy of 83.34% with a precision of 84.60%, a recall of 91.92%, and F1 score of 89.95% was achieved where the combination is No. 3 (6 × 6 cell and 4 × 4 block size) with LR, SVM, and No. 4 (6 × 6 cell size and 5 × 5 block size).  Figure 10(b) represents the results of HOG using SVM; an accuracy of 88.80% was reached with combination No. 3, while its precision, recall, and F1 score were 87.87%, 92.92%, and 89.85%, correspondingly. As shown in Figure 10(c), using RF, the highest accuracy of 79.13% with a precision of 80.28%, a recall of 81.03%, and F1 score of 79.09% was obtained.

#### 3.2.2. GLCM Results

**Figure 11:**
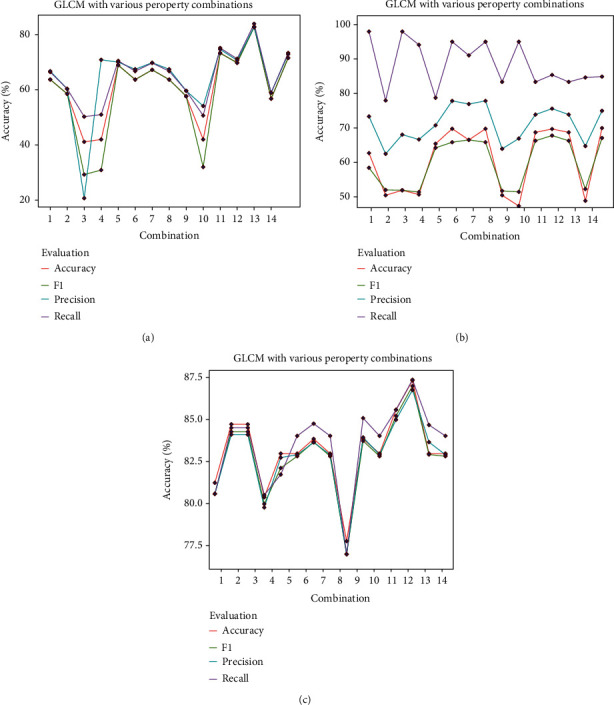
GLCM results with different property combinations.

For GLCM, four crucial properties were selected, including contrast, correlation, energy, and homogeneity as we mentioned in Subsection 2.5.2. All 15 combinations for these four properties were used to represent the texture feature of the histopathology images. The matrix property combinations are shown in Table 5, and its results are illustrated in Figure 11. As seen in Figure 11(a), using LR, the best accuracy of 73.04 with a precision of 74.38%, a recall of 74.90%, and a F1 score of 73.01% was obtained, where *contrast* + *correlation* + *energy* was used.  Figure 11(b) shows that with SVM, an accuracy of 71.79% with a precision of 67.88%, a recall of 97.04, and a F1 score of 67.88 was reached using *contrast* + *energy*. For RF, the highest accuracy of 82.60% was higher than that of LR and SVM, with a precision of 82.53%, a recall of 83.65%, and a F1 score of 82.44%, using *correlation* + *energy* + *homogeneity*.

#### 3.2.3. LBP Results

**Figure 12:**
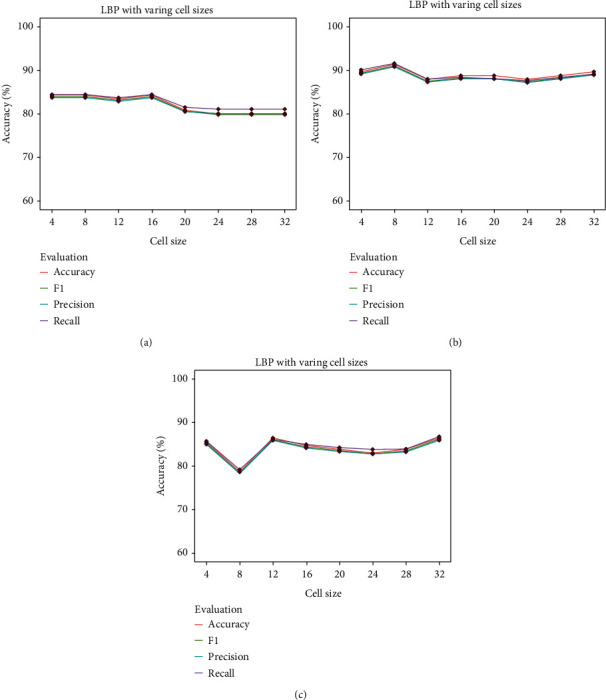
LBP results with varying cell sizes.

LBP as a higher-order statistical texture feature extraction method was used as the third extractor in the kidney histopathology images. The uniform LBP with 8 neighbors and radius 1 was used here since it has been proven to be compact and powerful [44]. We set the range of the cell size from 4 to 32. The results based on LBP with varying cell sizes using three traditional classifiers are represented in Figure 12. The highest accuracy using LR based on the LBP with cell size = 16 was 84.46% with a precision of 83.74%, a recall of 84.46%, and a F1 score of 83.99% (refer to Figure 12(a)). As shown in Figure 12(b), using SVM, an accuracy of 81.73% with a precision of 81.15%, a recall of 81.93%, and a F1 score of 81.37 was obtained, where the cell size = 8.  Figure 12(c) presents the results of using RF; the best accuracy with the cell size = 32 was 85.21% with a precision of 85.21%, a recall of 84.46%, and a F1 score of 83.99%.

### 3.3. Transform-Based Texture Feature Family Results

#### 3.3.1. Gabor Filter Results

**Figure 13:**
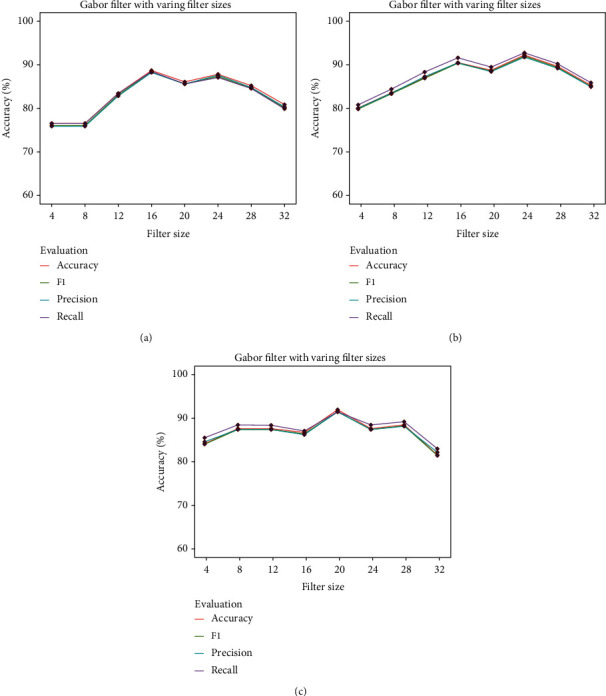
Gabor filter results with varying cell sizes.

The Gabor filter, as the most commonly used filter in pattern recognition was applied. Here, we varied the filter size for the Gabor filter; the range of the filter size is 4 : 4 : 32. The classification results by different classifiers based on the filter with an increasing filter size are illustrated in Figure 13. The highest accuracy obtained through LR was 88.69% with a precision of 88.23%, a recall of 88.46%, and a F1 score of 88.34% where the filter size = 16. As shown in Figure 13(b), using SVM, an accuracy of 86.08% with a precision of 85.59%, a recall of 86.59%, and a F1 score of 85.84% where the filter size = 24.  Figure 13(c) shows the results of using RF; an accuracy of 86.08% with a precision of 85.60%, a recall of 86.50%, and a F1score of 85.60% was obtained where the filter size = 20.

### 3.4. Model-Based Texture Feature Family Results

#### 3.4.1. MRF Results

**Figure 14:**
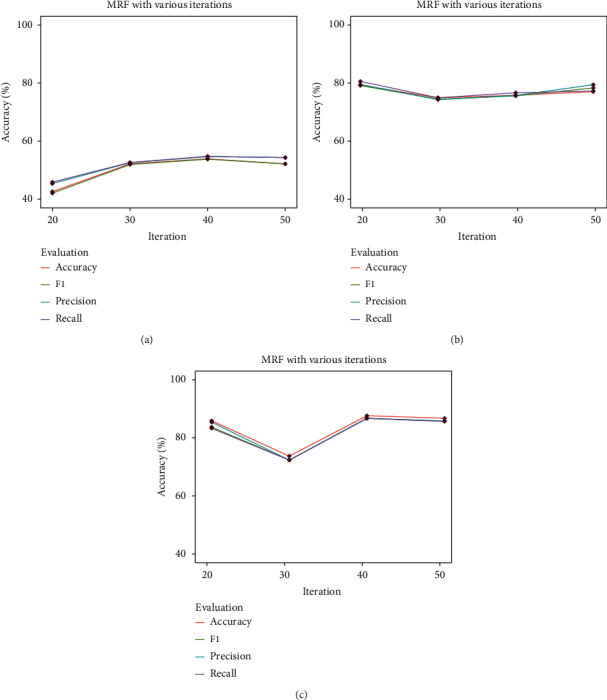
MRF results with various iterations.

MRF from the model-based texture feature family was the last extractor used in this paper. Here, MRF has an iteration parameter, ranging from 20 : 10 : 50, respectively (refer to Subsection 2.7.1).  Figure 14 shows the classification results using MRF while varying the number of iterations. As shown in Figure 14(a), using LR with an iteration of 40, an accuracy of 53.91% with a precision of 54.65%, a recall of 54.78%, and a F1 score of 53.78% was obtained, which is relatively low. The highest accuracy of 80.86% with a precision of 81.12%, a recall of 82.18%, and a F1 score of 80.75% using SVM was obtained, where the iteration was 50 (refer to Figure 14(b)). Using RF, an accuracy of 73.04% RF was obtained with a precision where the iteration is equal to 40 (refer to Figure 14(c)).

### 3.5. Proposed Model Results

In this subsection, we validated the proposed model on the medical dataset mentioned in Section 2.1. First, we fused both feature vectors by concatenating them. Later, three traditional classifiers, LR, SVM, and RF, were used to classify the data based on the fused vectors. In order to illustrate the effectiveness of the proposed model in terms of RC detection using histopathology images, we compared it with the deep learning model Alex-Net and the singular texture feature descriptors (HOG, GLCM, LBP, Gabor filter, and MRF); experiments results are shown in Figure 9 and Table 6. The proposed model reached the highest accuracy of 98.54% with the SVM classifier combining the Alex-Net and the Gabor filter features. As the results show, the proposed model reached an accuracy of 93.76% with Alex-Net fused with HOG, an accuracy of 94.52% with Alex-Net fused with GLCM, an accuracy of 93.45% with Alex-Net fused with LBP, and an accuracy of 97.39% with Alex-Net fused with MRF.

Due to the lack of equipment, most hospitals can only provide normal histopathology images with low lenses (at 100x magnification), where the quality of those images is much lower than a whole-slide image (WSI). In the future, we could explore the application of the proposed method on the WSI as literature. As a result, the accuracy of the classification is not as good as research that uses WSI [45, 46]. In the literature [29], GLCM with a SVM classifier was employed achieving an accuracy of 92.8% and GLCM with k-NN obtaining an accuracy of 91.65%. These results are remarkable compared with accuracies of 73.04%, 71.79%, and 82.60% we got while using GLCM only with LR, SVM, and RF. However, considering the large number of basic hospitals and the number of patients, we could build a much bigger dataset to verify the proposed method. Compared with the limited published datasets using WSI, normal histopathology images provided by basic hospitals might be more promising.

In the future, there are several options to explore regarding improving the accuracy of detecting RC using our method. One avenue is to vary the size of the dataset to establish the optimum quantity of images. Also, the impact of the hardware specifications should be considered, a dedicated machine versus setting minimum required specifications. Furthermore, we can consider more features like shape to describe the characteristics of histopathology images more comprehensively, before obtaining a better performance in RC detection, so that we can detect and diagnose RC early and effectively improve the survival and cure rate.

## 4. Conclusion

In this study, we proposed a classification model to detect renal cancer using a histopathology dataset by fusing the features from a deep learning model with the extracted texture feature descriptors. After the preprocessing of histopathology images including transformation and color normalization, we extracted deep features using the Alex-Net and texture features using five texture feature descriptors from three families separately to complement Alex-Net, then fused deep features and texture features for the classification of RC. To optimize the performance of the proposed method, various parameter(s) of each extractor were experimented. Experimental results validated that the proposed model outperformed the deep learning model or the singular texture feature descriptor; we extensively studied the effects of texture features to accomplish deep features. For the future work, we can apply the proposed model for different histopathology images dataset to optimize the performance.

## Figures and Tables

**Algorithm 1:**
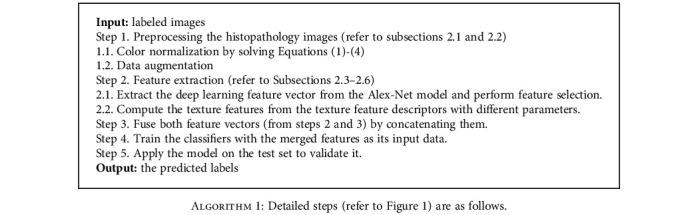
Detailed steps (refer to Figure 1) are as follows.

**Table 1:** List of the texture feature extractors.

Family	Method
Statistical	Gray-level cooccurrence matrix (GLCM) Histogram of oriented gradients (HOG) Local binary pattern (LBP)
Transform-based	Gaussian filter
Model-based	Markov random field (MRF)

**Table 2:** Statistics of the dataset.

Class	Images	Augmented Total	With augmentation	
			Training	Validation
RC	210	630	441	189
Healthy	140	560	392	168

**Table 3:** An example of kidney histopathology images' four properties for both a healthy and RC sample using GLCM.

Class	Contrast	Correlation	Energy	Homogeneity
Healthy	0.1246	0.7433	0.4055	0.9377
RC	0.1146	0.7556	0.4295	0.9427

**Table 4:** HOG cell size and block size combination list.

HOG cell size and block size	
(1) 6 × 6 cell size and 2 × 2 block size	(2) 6 × 6 cell size and 3 × 3 block size
(3) 6 × 6 cell size and 4 × 4 block size	(4) 6 × 6 cell size and 5 × 5 block size
(5) 8 × 8 cell size and 2 × 2 block size	(6) 8 × 8 cell size and 3×3 block size
(7) 8 × 8 cell size and 4 × 4 block size	(8) 8 × 8 cell size and 5 × 5 block size

**Table 5:** GLCM matrix property combination list.

Matrix property	
(1) Contrast	(2) Correlation
(3) Energy	(4) Homogeneity
(5) Contrast + correlation	(6) Contrast + energy
(7) Contrast + homogeneity	(8) Correlation + energy
(9) Correlation + homogeneity	(10) Energy + homogeneity
(11) Contrast + correlation + energy	(12) Contrast + correlation + homogeneity
(13) Contrast + energy + homogeneity	(14) Correlation + energy + homogeneity
(15) All	

**Table 6:** Summary of the best results.

Method	Classifier	Accuracy	Corresponding parameters (s)
Alex-Net		87.72%	180 epochs
HOG	SVM	88.80%	Cell size = 6 × 6, block size = 4 × 4
Alex − Net + HOG	SVM	93.76%	Cell size = 6 × 6, block size = 4 × 4
GLCM	RF	82.60%	Correlation + energy + homogeneity
Alex − Net + GLCM	RF	94.52%	Correlation + energy + homogeneity
LBP	RF	85.21%	Cell size = 12
Alex − Net + LBP	RF	93.45%	Cell size = 12
Gabor filter	LR	88.69%	Cell size = 16
Alex − Net + Gabor filter	LR	98.54%	Cell size = 16
MRF	SVM	80.86%	Iteration = 50
Alex − Net + MRF	SVM	97.39%	Iteration = 50

## Data Availability

The data used to support the findings of this study are available from the corresponding author upon request.

## References

[1] Gurcan M. N., Boucheron L. E., Can A., Madabhushi A., Rajpoot N. M., Yener B. (2009). Histopathological image analysis: a review. *IEEE Reviews in Biomedical Engineering*.

[2] Veta M., Pluim J. P. W., van Diest P. J., Viergever M. A. (2014). Breast cancer histopathology image analysis: a review. *IEEE Transactions on Biomedical Engineering*.

[3] Märkl B., Füzesi L., Huss R., Bauer S., Schaller T. (2021). Number of pathologists in Germany: comparison with European countries, USA, and Canada. *Virchows Archiv*.

[4] Fuchs T. J., Buhmann J. M. (2011). Computational pathology: challenges and promises for tissue analysis. *Computerized Medical Imaging and Graphics*.

[5] Ghaznavi F., Evans A., Madabhushi A., Feldman M. (2013). Digital imaging in pathology: whole-slide imaging and beyond. *Annual Review of Pathology: Mechanisms of Disease*.

[6] Coudray N., Ocampo P. S., Sakellaropoulos T. (2018). Classification and mutation prediction from non-small cell lung cancer histopathology images using deep learning. *Nature Medicine*.

[7] Tabibu S., Vinod P. K., Jawahar C. V. (2019). Pan-renal cell carcinoma classification and survival prediction from histopathology images using deep learning. *Scientific Reports*.

[8] Key Statistics About Kidney Cancer. https://www.cancer.org/cancer/kidney-cancer/about/key-statistics.html.

[9] Fenstermaker M., Tomlins S. A., Singh K., Wiens J., Morgan T. M. (2020). Development and validation of a deep-learning model to assist with renal cell carcinoma histopathologic interpretation. *Urology*.

[10] Armi L., Fekri Ershad S. (2019). Texture image analysis and texture classification methods - a review. http://arxiv.org/abs/1904.06554.

[11] Fekri-Ershad S. (2019). Pap smear classification using combination of global significant value, texture statistical features and time series features. *Multimedia Tools and Applications*.

[12] Sharma N., Aggarwal L. M. (2010). Automated medical image segmentation techniques. *Journal of medical physics/Association of Medical Physicists of India*.

[13] Lan R., Zhong S., Liu Z., Shi Z., Luo X. (2018). A simple texture feature for retrieval of medical images. *Multimedia Tools and Applications*.

[14] Alhindi T. J., Kalra S., Ng K. H., Afrin A., Tizhoosh H. R. Comparing LBP, HOG and deep features for classification of histopathology images.

[15] Korkmaz S. A., Akçiçek A., Bínol H., Korkmaz M. F. Recognition of the stomach cancer images with probabilistic HOG feature vector histograms by using HOG features.

[16] Mercan C., Aksoy S., Mercan E., Shapiro L. G., Weaver D. L., Elmore J. G. (2018). Multi-instance multi-label learning for multi-class classification of whole slide breast histopathology images. *IEEE Transactions on Medical Imaging*.

[17] Vahadane A., Peng T., Sethi A. (2016). Structure-preserving color normalization and sparse stain separation for histological images. *IEEE Transactions on Medical Imaging*.

[18] Krizhevsky A., Sutskever I., Hinton G. E. (2012). ImageNet classification with deep convolutional neural networks. *Advances in Neural Information Processing Systems*.

[19] Nawaz W., Ahmed S., Tahir M., Khan H. A. (2018). Classification of breast cancer histology images using ALEXNET. *International conference image analysis and recognition*.

[20] Chen J., Wan Z., Zhang J. (2021). Medical image segmentation and reconstruction of prostate tumor based on 3D AlexNet. *Computer Methods and Programs in Biomedicine*.

[21] Titoriya A., Sachdeva S. Breast cancer histopathology image classification using AlexNet.

[22] Spanhol F. A., Oliveira L. S., Petitjean C., Heutte L. (2016). A dataset for breast cancer histopathological image classification. *IEEE Transactions on Biomedical Engineering*.

[23] Humeau-Heurtier A. (2019). Texture feature extraction methods: a survey. *IEEE Access*.

[24] Castellano G., Bonilha L., Li L. M., Cendes F. (2004). Texture analysis of medical images. *Clinical Radiology*.

[25] Ismael M. R., Abdel-Qader I. Brain tumor classification via statistical features and back-propagation neural network.

[26] Dalal N., Triggs B. Histograms of oriented gradients for human detection.

[27] Huu P. N., Phung Ngoc T. (2021). Hand gesture recognition algorithm using SVM and HOG model for control of robotic system. *Journal of Robotics*.

[28] Feng J., Yang Y.-J. (2007). Study of texture images extraction based on gray level co-occurence matrix. *Beijing Surveying and Mapping*.

[29] Öztürk Ş., Akdemir B. (2018). Application of feature extraction and classification methods for histopathological image using GLCM, LBP, LBGLCM, GLRLM and SFTA. *Procedia Computer Science*.

[30] Garg M., Dhiman G. (2021). A novel content-based image retrieval approach for classification using GLCM features and texture fused LBP variants. *Neural Computing and Applications*.

[31] Verma M., Raman B. (2015). Center symmetric local binary co-occurrence pattern for texture, face and bio- medical image retrieval. *Journal of Visual Communication and Image Representation*.

[32] A comparative study of texture measures with classification based on featured distributions ScienceDirect https://www.sciencedirect.com/science/article/pii/0031320395000674.

[33] Ahonen T., Hadid A., Pietikainen M. (2006). Face description with local binary patterns: application to face recognition. *IEEE Transactions on Pattern Analysis and Machine Intelligence*.

[34] Jabri S., Saidallah M., El Alaoui A. E. B., El Fergougui A. Moving vehicle detection using Haar-like, LBP and a machine learning Adaboost algorithm.

[35] Lee C.-J., Wang S.-D. (1999). Fingerprint feature extraction using Gabor filters. *Electronics Letters*.

[36] Ahonen T., Hadid A., Pietikäinen M. (2004). Face recognition with local binary patterns. *European conference on computer vision*.

[37] Dobrisek: Face recognition in the wild with the Google Scholar https://scholar.google.com/scholar_lookup?title=Face+recognition+in+the+wild+with+the+probabilistic+gabor-fisher+classifier&conference=Proceedings+of+the+2015+11th+IEEE+International+Conference+and+Workshops+on+Automatic+Face+and+Gesture+Recognition+.

[38] Cross G. R., Jain A. K. (1983). Markov random field texture models. *IEEE Transactions on Pattern Analysis and Machine Intelligence*.

[39] Ashraf A. B., Gavenonis S. C., Daye D., Mies C., Rosen M. A., Kontos D. (2013). A multichannel Markov random field framework for tumor segmentation with an application to classification of gene expression-based breast cancer recurrence risk. *IEEE Transactions on Medical Imaging*.

[40] Rastghalam R., Pourghassem H. (2016). Breast cancer detection using MRF-based probable texture feature and decision- level fusion-based classification using HMM on thermography images. *Pattern Recognition*.

[41] Xu Y., Jia Z., Wang L. B. (2017). Large scale tissue histopathology image classification, segmentation, and visualization via deep convolutional activation features. *BMC Bioinformatics*.

[42] He S., Ruan J., Long Y. Combining deep learning with traditional features for classification and segmentation of pathological images of breast cancer.

[43] Tan H., Yang B., Ma Z. (2014). Face recognition based on the fusion of global and local HOG features of face images. *IET Computer Vision*.

[44] Dinesh Kumar M., Babaie M., Zhu S., Kalra S., Tizhoosh H. R. A comparative study of CNN, BoVW and LBP for classification of histopathological images.

[45] Xie J., Liu R., Luttrell J., Zhang C. (2019). Deep learning based analysis of histopathological images of breast cancer. *Frontiers in Genetics*.

[46] Jiang Y., Chen L., Zhang H., Xiao X. (2019). Breast cancer histopathological image classification using convolutional neural networks with small SE-ResNet module. *PLoS One*.

